# Pu-erh Tea Reduces Nitric Oxide Levels in Rats by Inhibiting Inducible Nitric Oxide Synthase Expression through Toll-Like Receptor 4

**DOI:** 10.3390/ijms13067174

**Published:** 2012-06-11

**Authors:** Yang Xu, Guan Wang, Chunjie Li, Min Zhang, Hang Zhao, Jun Sheng, Wei Shi

**Affiliations:** 1 Key Laboratory for Molecular Enzymology and Engineering, the Ministry of Education, College of Life Science, Jilin University, Changchun 130012, China; E-Mails: xuyang759@yahoo.com.cn (Y.X.); wxkzbwg@163.com (G.W.); lichunjie0821@163.com (C.L.); zhangmin2584652@yahoo.com.cn (M.Z.); zhzky@163.com (H.Z.); 2 Yunnan Research Centre for Advance Tea Processing, Yunnan Agricultural University, Kunming 650201, China

**Keywords:** pu-erh tea, nitric oxide, inducible nitric oxide synthase, polysaccharides, Toll-like receptor 4

## Abstract

Pu-erh tea undergoes a unique fermentation process and contains theabrownins, polysaccharides and caffeine; although it is unclear about which component is associated with the down regulation of nitric oxide levels or how this process is mediated. To address this question we examined the effects of pu-erh tea on nitric oxide synthase (NOS) genes. Cohorts of rats were separately given four-week treatments of water as control, pu-erh tea, or the tea components: theabrownins, caffeine or polysaccharides. Five experimental groups were injected with lipopolysaccharides (LPS) to induce nitric oxide (NO) production, while the corresponding five control groups were injected with saline as a negative control. The serum and liver NO concentrations were examined and the NOS expression of both mRNA and protein was measured in liver. The results showed that the rats which were fed pu-erh tea or polysaccharides had lower levels of NO which corresponded with the down-regulation of inducible nitric oxide synthase (iNOS) expression. We further demonstrate that this effect is mediated through reduction of Toll-like receptor 4 (TLR4) signaling. Thus we find that the polysaccharide components in pu-erh tea reduce NO levels in an animal model by inhibiting the iNOS expression via signaling through TLR4.

## 1. Introduction

Nitric oxide (NO) is a free radical which is produced by nitric oxide synthase (NOS) in the body. Three NOS isoforms have been identified, neural nitric oxide synthase (nNOS), endothelial nitric oxide synthase (eNOS) and inducible nitric oxide synthase (iNOS). nNOS and eNOS are constitutive, whereas iNOS is inducible in response to various stimuli, such as lipopolysaccharides (LPS) which can activate Toll-like receptor 4 (TLR4) signal pathway [1,2]. NO is mainly produced by eNOS and iNOS in the liver [3,4]. A well-balanced level of NO is important for various physiological processes, such as mediating macrophage cytotoxicity, regulating blood pressure, and neurotransmission [5,6]. However, overproduction of NO has been implicated in causing tissue damage, leading to conditions such as cancer, diabetes, renal disease and cardiovascular disease [7–9]. Therefore excess NO generation should be avoided and foods that aid in inhibiting the pro-inflammatory NF-κB-mediated iNOS activity may offer a potential health benefit.

The popular beverage pu-erh tea (written as pu-erh, pu-er, or puer) is mainly produced in the Yunnan province of China and consumed widely all over the world. In recent years, studies of the possible health benefits on pu-erh tea have shown a wide range of biological effects, such as antioxidative [10,11], antiobesity [12,13], antihyperlipidemic [14], antimutagenic [15], and antimicrobial [15]. The chemical components and properties of teas vary greatly due to the season when the tealeaves are harvested and the fermentation methods used. Generally, green tea undergoes a short fermentation and contains more reduced polyphenols, such as catechins, whereas pu-erh tea has a longer fermentation process resulting in more polymeric polyphenols called theabrownins [16–18]. Additional components of pu-erh tea are also indispensable for its health benefit, such as polysaccharides and caffeine [19]. Our previous work found that pu-erh tea could inhibit the RAW 246.7 cells to generate NO [20]. However, the effect of polysaccharides on inhibiting NO production and the causative mechanism remain unclear.

In this study, we demonstrated for the first time that the unique polysaccharides in pu-erh tea exerted an inhibitory effect on iNOS expression by reducing the activity of TLR4, which is the initiative molecule of the iNOS expression signal pathway. Our work provides a molecular explanation for the ability of pu-erh tea to reduce levels of the free radical NO.

## 2. Results and Discussion

### 2.1. Main Contents in Pu-erh Tea

In the previous study, we analyzed and reported the main contents in pu-erh tea [20]. Approximately one third (33.13 ± 3.18%) of pu-erh tea consisted of polyphenols, of which theabrownins represented a significant fraction (7.32–10.50%). The amounts of polysaccharides (4.81 ± 0.13%) and caffeine (9.31 ± 0.09%) were also determined in pu-erh tea, also as main components of pu-erh tea. The results reflected the abundance of theabrownins, polysaccharides and caffeine in pu-erh tea. Next we fed rats each a separate fraction as well as pu-erh tea, to identify which was the main assistant of scavenging NO.

### 2.2. NO Level in Serum

**Figure 1:**
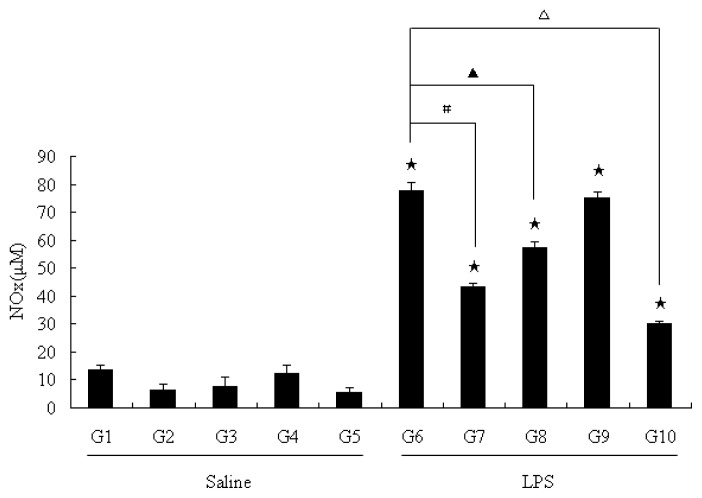
NOx (nitrite/nitrate) productions in rat serum. NO level in rat serum was determined by measuring total NOx in serum. The results were expressed as percentage of control and were represented by mean ± SE determined from three independent experiments. All *p* values were for comparisons between control groups and experiment groups. ★ indicated *p* < 0.05 of each LPS treated groups with one kind of drink versus its saline treated groups as control respectively. #, ▲ or △ indicated *p* < 0.05 between G6 and one of other groups that were treated with LPS. LPS, lipopolysaccharides; NOx, nitrite/nitrate. Details about groups are mentioned in Materials and Methods.

An inhibitory effect of pu-erh tea on the LPS-induced iNOS expression in macrophages has been reported in cultured macrophages [21], however there were no *in vivo* animal studies examining the inhibitory role of pu-erh tea on iNOS, and the mechanism remains unclear. Thus, we designed the following animal experiments to study the effect of pu-erh tea. In each of the ten groups, the rats increased their weight at a constant rate. There were no differences in weight gain among the groups (data not shown). Nitric oxide production was estimated in serum from rats of group1 (water + saline) that served as controls by measuring the stable metabolite NOx (nitrite/nitrate). A normal production of NOx (13.58 ± 1.83 μM, *n* = 10) was found in these samples (Figure 1). LPS was found to induce a significant increase of NOx in serum (G6–G10 in Figure 1). Among the groups of the rats which were not injected with LPS, the pu-erh tea group (G2), the theabrownins group (G3) and the polysaccharides group (G5) slightly reduced the NO level in serum, compared with the water group (G1). In LPS-treated groups, NO level of G7, G8 and G10 had a significant decline compared with the water consumed LPS group (G6) as shown in Figure 1. These results indicated that pu-erh tea feeding can reduce serum NO levels, possibly through the theabrownins and/or polysaccharide components in pu-erh tea.

### 2.3. NO Level in Liver Homogenates

**Figure 2:**
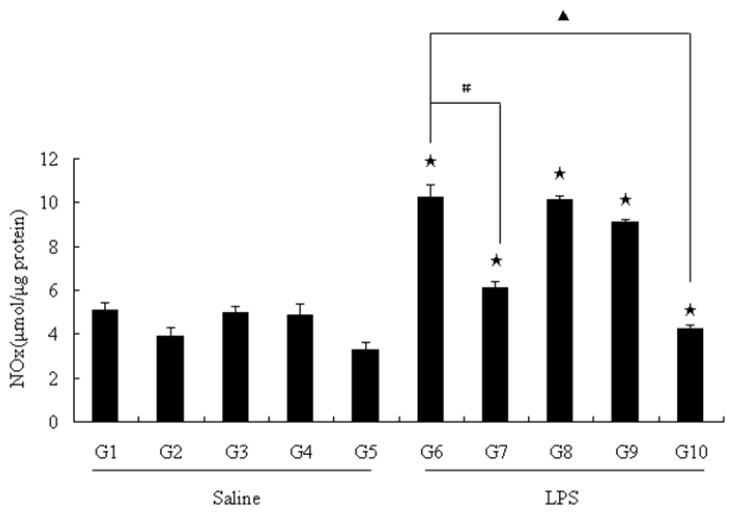
NOx productions in rat liver. NO level in rat liver was determined by measuring total NOx in the liver homogenates. The results were expressed as percentage of control and were represented by mean ± SE determined from three independent experiments. All *p* values were for comparisons between control groups and experiment groups. ★ indicated *p* < 0.05 of each LPS-treated group with one kind of drink *versus* its saline-treated group as control respectively. # or ▲ indicated *p* < 0.05 between G6 and one of other groups that were treated with LPS. LPS, lipopolysaccharides; NOx, nitrite/nitrate. Details about groups are mentioned in Materials and Methods.

In liver homogenates of the rats, the metabolic level of NO was shown as group1 (water + saline) in Figure 2. A basal production of NOx was about 5.12 ± 0.33 μmol/μg of protein in liver. In the groups of rats that were administered with saline as controls, NO production in the liver was slightly inhibited by being fed with pu-erh tea and tea polysaccharides. In Figure 2, the water + LPS group (G6) had a NOx concentration in liver homogenates of 10.25 ± 0.56 μmol/μg of protein. Interestingly, the groups of rats that were fed theabrownins did not significantly reduce liver NO levels as we previously found in the serum. Only the pu-erh tea group (G7 6.11 ± 0.3 μmol/μg of protein) and the polysaccharides group (G10 4.22 ± 0.19 μmol/μg of protein) helped the rats to significantly reduce the level of NO from the liver. Figure 1 showed that the NO in serum was significantly reduced by feeding pu-erh tea, theabrownins and polysaccharides (G7, G8, G10). However, in liver, the theabrownins feeding group (G8) failed to reduce NO production, as shown in Figure 2. One possible explanation is that the theabrownins directly reacted with NO in the serum because of their ability as antioxidants as a kind of polyphenol, which scavenged the dissociative NO in the serum. However, polysaccharides could not scavenge the NO by reacting with NO directly. It likely reduced the NO production by regulating the expression of the enzymes in liver, which was tested by RT-PCR and Western blot subsequently.

### 2.4. NOS mRNA Level in Liver

**Figure 3:**
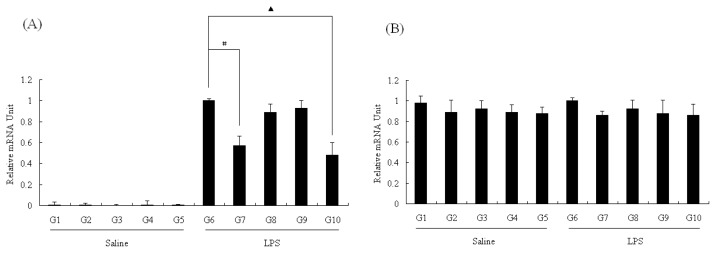
NOS mRNA level in rat liver. (**A**) Relative mRNA level of iNOS was measured by real-time PCR; (**B**) Relative mRNA level of eNOS was measured by real-time PCR. The results were expressed as percentage of control (G6) and were represented by mean ± SE determined from three independent experiments. All *p* values were for comparisons between control groups and experiment groups. # or indicated *p* < 0.05 between G6 and one of other groups that were treated with LPS. LPS, lipopolysaccharides; NOx, nitrite/nitrate. Details about groups are mentioned in Materials and Methods.

The liver is an important organ that produces endogenous NO in the body [22]. Thus, we tested the NOS expression in the liver to investigate whether there was inhibition of NOS expression by pu-erh tea that attenuated NO production in rats. To investigate whether the effect of pu-erh tea on NO production in rat liver tissue was due to the modulation of NOS expression, liver homogenates were examined using RT-PCR. Both eNOS and iNOS are expressed in the liver and were assayed using the primers listed in Table 1. As shown in Figure 3A, treatment with LPS increased iNOS mRNA levels in the liver, while there was no change in eNOS levels in the control or LPS treated groups in Figure 3B. Compared with G6 (water + LPS) as control whose relative mRNA value was 1 in Figure 3A, the rats fed with pu-erh tea or tea polysaccharides for four weeks had less iNOS mRNA. The relative mRNA units of these two groups (G7 and G10) are respectively 0.57 ± 0.09 and 0.48 ± 0.12. These results were in agreement with the data presented in Figure 2, which implied that pu-erh tea inhibited NO production by strongly suppressing iNOS expression at the transcriptional level, and one of its components, polysaccharides, is likely to be the active ingredient.

### 2.5. NOS Protein Expression in Liver

**Figure 4:**
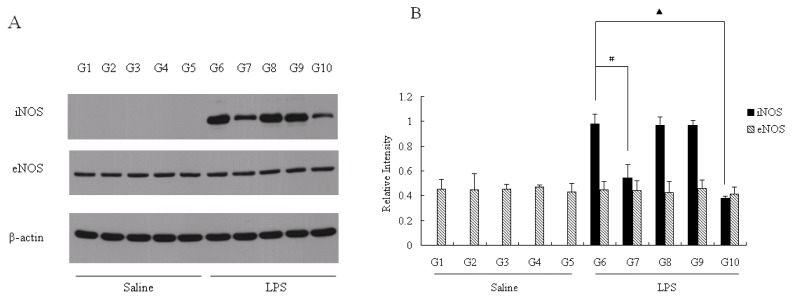
NOS protein expression in rat liver. (**A**) The protein expression of iNOS, eNOS and β-actin as control was measured by western blot; (**B**) The protein level of iNOS and eNOS was quantified versus β-actin as control by using software, Image J. The results were expressed as percentage of control and were represented by mean ± SE determined from three independent experiments. All *p* values were for comparisons between control groups and experiment groups. # or ▲ indicated *p* < 0.05 between G6 and one of the other groups that were treated with LPS. LPS, lipopolysaccharides; NOx, nitrite/nitrate. Details about groups are mentioned in Materials and Methods.

Given the confirmation that pu-erh tea and its polysaccharides could help rats to reduce NO production in liver via down-regulating iNOS expression at mRNA level, we next focused on protein levels of iNOS and eNOS in the liver. Figure 4A shows that if the rats were not injected with LPS, iNOS protein was undetectable, whereas the bands of iNOS were obvious of the LPS groups (G6–G10). Consistent with mRNA expression in Figure 3, the level of eNOS proteins in the ten groups was not affected by the injection of LPS nor regulated by pu-erh tea feeding. Quantitation of the normalized iNOS and eNOS western blot levels using Image J software (version 1.45; National Institutes of Health (NIH): Bethesda, MD, USA, 2011) reveals that the G7 iNOS protein level was reduced by almost half of the control group (G6), and G10 iNOS expression was nearly one third of G6. These results were consistent with Figure 2, which supported our hypothesis that polysaccharides act mainly through inhibiting iNOS expression. Notably, the iNOS mRNA expression of G7 or G10 was not inhibited as strongly as their protein levels, comparing Figure 3 with Figure 4. This suggests another possible mechanism in addition to transcriptional suppression, possibly during the translation or processing of iNOS protein, might also be affected by pu-erh tea.

### 2.6. Expression and Activities of iNOS Expression Signal Pathway

**Figure 5:**
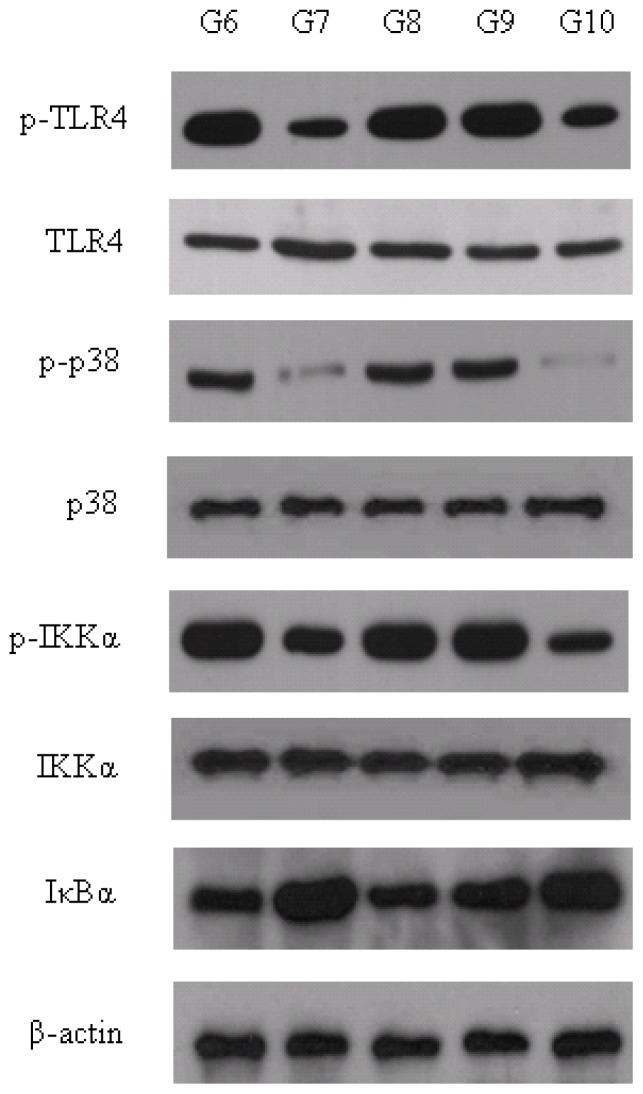
Expression and activities of iNOS expression signal pathway. The amount of total and phosphorylated TLR4, p38 and IKKα were measured by western blot (p, phosphorylated). Levels of IκBα, which is under the control of ubiquitination and degradation via IKKα, was compared to beta-actin loading control. Details about groups are mentioned in Materials and Methods.

It is known that after the cells are stimulated by LPS, iNOS expression will be switched on via a specific signal pathway. LPS binds to CD14, a high-affinity LPS receptor. TLR4 interacts with this CD14-LPS complex, and then activates two intracellular signaling cascades, p38 and IKKα, which are upstream of iNOS transcription. At resting state before IKKα is activated, NFκB is bound by IκBα to repress signaling. Activation of IKKα stimulates IκBα phosphorylation, which triggers the ubiquitination and subsequent degradation of IκBα, and results in available NFκB. As a result, the transcription factor NFκB, together with AP1, which is activated by phosphorylated p38, initiates the transcription of iNOS gene [23–25]. To better understand the mechanism by which pu-erh tea and polysaccharides inhibit the expression of iNOS, we tested the expression and activities of several key proteins in the iNOS expression signal pathway. As shown in Figure 5, the cell surface receptor TLR4 and its downstream molecules, p38 and IKKα, were less phosphorylated in liver than pu-erh tea and polysaccharides feeding groups. Meanwhile, as the substrate of the IKKα kinase, more IκBα were under ubiquitination, resulting in more IκBα degradation. These results indicate that the activity of the iNOS expression signaling was restrained by polysaccharides from TLR4 which is the initiation of this pathway. We speculate that the polysaccharides of pu-erh tea may have similar binding epitopes as LPS, which resulted in polysaccharides competitively inhibiting the binding between LPS and CD14, further reducing the activity of TLR4.

## 3. Experimental Section

### 3.1. Materials

TRIzol reagent was obtained from TaKaRa (Tokyo, Japan), First-Strand cDNA Synthesis Kit (Fermentas, Glen Burnie, MD, USA) and the SYBR Universal PCR Master Mix (Tiangen, Beijing, China) were used in the present research. PCR primers used in this study were synthesized by Sangon (Shanghai, China). The monoclonal antibodies used in the study were bought from Santa Cruz Biotechnology (Santa Cruz, CA, USA) and Cell Signaling Technology (Danvers, MA, USA). Lipopolysaccharide (LPS; Escherichia coli serotype O111:B4) and all other chemicals employed in this study were of analytical grade and were purchased from Sigma Chemical Co. (St. Louis, MO, USA). The pu-erh tea extract, polysaccharides, theabrownins and caffeine were made and provided by China Academy of Pu-erh Tea Research (Pu Erh, Yunnan, China).

### 3.2. Determination of Polyphenols, Polysaccharides, and Caffeine Content in Concentrated Pu-erh Tea Extracts

Polyphenol content analysis was performed under the guidelines of national standards using the ferrous tartrate method [26,27]. Briefly, the tea extraction solution, buffer solution and ferrous tartrate tetrahydrate solution were mixed in 25-mL capacity bottle. Absorbance (A) at 540 nm with a 10 mm quartz cell was used to calculate the extraction of tea polyphenols. Polysaccharides were quantitated using the anthrone-sulfuric acid method using glucose as standard as described [28]. A standard curve was generated with glucan, which was linear between the concentration range of 5 and 30 μg. The calibration curve equation was y = 0.063× + 0.0579 and had a correlation coefficient of *R*^2^ = 0.9957. Caffeine was quantitated using the lead subacetate method [27]. A standard curve was generated with caffeine, which was linear between the concentration range of 50 and 300 μg. The calibration curve equation was y = 62.911× + 0.0058 and had a correlation coefficient of *R*^2^ = 0.9997.

### 3.3. Experimental Animals

Male Sprague-Dawley rats weighing 280 ± 20 g were obtained from the Animal Resource Center of Jilin University. The researches on rats were approved by the Animal Committee of Jilin University. They were kept under controlled temperature (22–24 °C) and illumination (12-h light cycle starting at 7 a.m.). Animals were maintained on standard laboratory chow and water. After acclimation for 6–7 days, animals were randomly divided into 10 groups (10 animals each):

1. G1 (group1): the group consumed water, which was injected with saline;
2. G2 (group2): the group consumed pu-erh tea aqueous extracts (250 mg/kg/day) dissolved in water, which were injected with saline;
3. G3 (group3): the group consumed pu-erh tea theabrownins (50 mg/kg/day) dissolved in water, which were injected with saline;
4. G4 (group4): the group consumed pu-erh tea caffeine (50 mg/kg/day) dissolved in water, which was injected with saline;
5. G5 (group5): the group consumed pu-erh tea polysaccharides (50 mg/kg/day) dissolved in water, which were injected with saline;
6. G6 (group6): the group consumed water, which was injected with LPS;
7. G7 (group7): the group consumed pu-erh tea aqueous extracts (250 mg/kg/day) dissolved in water, which were injected with LPS;
8. G8 (group8): the group consumed pu-erh tea theabrownins (50 mg/kg/day) dissolved in water, which were injected with LPS;
9. G9 (group9): the group consumed pu-erh tea caffeine (50 mg/kg/day) dissolved in water, which was injected with LPS;
10. G10 (group10): the group consumed pu-erh tea polysaccharides (50 mg/kg/day) dissolved in water, which were injected with LPS.

Pu-erh tea aqueous extracts were dissolved in distilled water to make an aqueous solution of 5 mg/mL. Theabrownins, caffeine and polysaccharides were dissolved into distilled water to make aqueous solutions of 1 mg/mL respectively. The rats were administrated separately by gavage feeding three times per day according to the amount mentioned above. After four-week feeding of each group’s extracts mentioned above, treatment rats were administered either 5 mg/kg, i.p., LPS dissolved in sterile saline. Control rats were injected with an equivalent volume of sterile saline (5 mL/kg). Six hours later, animals were sacrificed, having first been subjected to blood and liver sampling.

### 3.4. NO Assay

The blood was collected in heparinized tubes. The liver samples were homogenized in cold 0.9% saline. The homogenates were then centrifuged at 10,000 r/min for 5 min at 4 °C, the supernatant was taken for NO assay and total protein determination. Nitric oxide production was determined by measuring in serum samples and liver homogenates total NOx, the stable end products, using a modification of Griess’s reaction, as previously described [20].

### 3.5. Protein Assay

Homogenates of liver tissue were examined for estimation of total protein per well using the Bio-Rad protein microassay. The protein assay was based on Bradford’s dye-binding procedure [29]. Briefly, known concentrations of bovine serum albumin were used as standard curves. Two hundred microliters of sample or standard and 50 μL of Bio-Rad protein assay was added per well in a 96-well microtiter plate and protein was measured with a microplate reader.

### 3.6. RT-PCR

Total RNA was isolated from approximately 50–100 mg snap-frozen liver tissue using the TRIzol protocol as suggested by the supplier. The purity of total RNA was determined by the ratio at 260 nm and 280 nm absorbance. 2 mg of total RNA was subjected to reverse transcription using RevertAid™ First-Strand cDNA Synthesis Kit with oligo-dT. 1 μL of the cDNA solution was used for real-time PCR. The genes were amplified in a 25 μL reaction on Bio-Rad Opticon Monitor (Applied Biosystems). The conditions comprised an initial denaturation step at 95 °C for 5 min, followed by 40 cycles each of 95 °C for 30 s, 60 °C for 15 s and 72 °C for 1 min, before being subjected to melting curve analysis. Amplified products were further verified by automated cycle sequencing. The 2^−ΔΔCT^ method [30,31] was used for calculating the relative expression level. The primer sequences of the proteins were shown in Table 1.

### 3.7. Western Blot

Homogenates of liver tissue were centrifuged at 12,000 g for 20 min at 4 °C. A total of 30 μg protein from each well was separated by 8% (or 10%) SDS-PAGE and transferred to polyvinylidene difluoride (PVDF) membranes. The membranes were blocked at room temperature for 1 h with 5% skim milk in tris-buffered saline with 0.1% Tween20 (TBST), and followed by incubation with primary antibody diluted with 5% skim milk in TBST at 4 °C overnight and then incubated with secondary antibody conjugated to alkaline phosphatase and detected using the ECL chemiluminescent system. Loading differences were normalized using a monoclonal β-actin antibody.

### 3.8. Statistical Analysis

The results were presented as mean ± SE. One-way ANOVA was used for a statistical comparison. (A probability level of 5%, *p* < 0.05 was considered statistically significant).

## 4. Conclusions

In conclusion, our findings suggest that drinking pu-erh tea could assist the body to reduce NO generation. The polysaccharides in pu-erh tea played the main role in this benefit by inhibiting iNOS expression. These results bring the promise of finding new drugs against diseases caused by NO overproduction. However, the exact mechanism by which the pu-erh tea polysaccharides inhibit the activity of TLR4 still needs to be confirmed. Thus, more work should be done to find out how the iNOS expression signal pathway is affected by pu-erh tea and its components.

## Figures and Tables

**Table 1:** Primer sequences used for reverse RT-PCR.

Gene	Primers	Tm
iNOS	Sense: 5′-CAGATCCCGAAACGCTACAC-3′	60.05 °C
	Antisense: 5′-TGCGGCTGGACTTCTCACT-3′	59.5 °C
eNOS	Sense: 5′-CTGCTGCCCCAGATATCTTC-3′	60.5 °C
	Antisense: 5′-CAGGTACTGCAGTCCCTCCT-3′	62.5 °C
β-actin	Sense: 5′-AACCCTAAGGCCAACCGTGAAAAG-3′	59.4 °C
	Antisense: 5′-TCATGAGGTAGTCTGTCAGGT-3′	61.37 °C

## References

[1] Zhang Y.Q., Mao Z., Zheng Y.L., Han B.P., Chen L.T., Li J., Li F. (2008). Elevation of inducible nitric oxide synthase and cyclooxygenase-2 expression in the mouse brain after chronic nonylphenol exposure. Int. J. Mol. Sci.

[2] Medvedev A.E., Piao W., Shoenfelt J., Rhee S.H., Chen H., Basu S., Wahl L.M., Fenton M.J., Vogel S.N. (2007). Role of TLR4 tyrosine phosphorylation in signal transduction and endotoxin tolerance. J. Biol. Chem.

[3] Chen T., Zamora R., Zuckerbraun B., Billiar T.R. (2003). Role of nitric oxide in liver injury. Curr. Mol. Med.

[4] McNaughton L., Puttagunta L., Martinez-Cuesta M.A., Kneteman N., Mayers I., Moqbel R., Hamid Q., Radomski M.W. (2002). Distribution of nitric oxide synthase in normal and cirrhotic human liver. Proc. Natl. Acad. Sci. USA.

[5] Mayer B., Hemmens B. (1997). Biosynthesis and action of nitric oxide in mammalian cell. Trends Biochem. Sci.

[6] Lin Y.S., Tsai Y.J., Tsay J.S., Lin J.K. (2003). Factors affecting the levels of tea polyphenols and caffeine in tea leaves. J. Agric. Food Chem.

[7] Beckman J.S., Koppenol W.H. (1996). Nitric oxide, superoxide, and peroxynitrite: The good, the bad, and ugly. Am. J. Physiol.

[8] Cooke J.P., Dzau V.J. (1997). Nitric oxide synthase: Role in the genesis of vascular disease. Annu. Rev. Med.

[9] Yermilov V., Rubio J., Becchi M., Friesen M.D., Pignatelli B., Ohshima H. (1996). Formation of 8-nitroguanine by the reaction of guanine with peroxynitrite *in vitro*. Carcinogenesis.

[10] Jie G., Lin Z., Zhang L., Lv H., He P., Zhao B. (2006). Free radical scavenging effect of pu-erh tea extracts and their protective effect on oxidative damage in human fibroblast cells. J. Agric. Food Chem.

[11] Luis M., Irene C., Jose A.B., Pedro R. (2006). Antioxidant effect of rosemary, borage, green tea, pu-erh tea and ascorbic acid on fresh pork sausages packaged in a modified atmosphere: Influence of the presence of sodium chloride. J. Agric. Food Chem.

[12] Jeng K.C., Chen C.S., Fang Y.P., Hou R.C., Chen Y.S. (2007). Effect of microbial fermentation on content of statin, GABA, and polyphones in pu-erh tea. J. Agric. Food Chem.

[13] Sano M., Takenaka Y., Kojima R., Saito S., Tomita I., Katou M., Shibuya S. (1986). Effects of pu-erh tea on lipid metabolism in rats. Chem. Pharm. Bull. (Tokyo).

[14] Cao Z.H., Gu D.H., Lin Q.Y., Xu Z.Q., Huang Q.C., Rao H., Liu E.W., Jia J.J., Ge C.R. (2011). Effect of pu-erh tea on body fat and lipid profiles in rats with diet-induced obesity. Phytother. Res.

[15] Wu S.C., Yen C.Y., Wang B.S., Chih C.K., Yen W.J., Chang L.W., Duh P.D. (2007). Antimutagenic and antimicrobial activities of pu-erh tea. Food Sci. Technol.

[16] Yao L.H., Liu X., Jiang Y.M., Nola C., Bruce D., Riantong S., Nivedita D., Xu Y. (2006). Compositional analysis of teas from Australian supermarkets. Food Chem.

[17] Gong J., Peng C., Chen T., Gao B., Zhou H. (2010). Effects of theabrownin from pu-erh tea on the metabolism of serum lipids in rats: Mechanism of action. J. Food Sci.

[18] Wang D., Xiao R., Hu X., Xu K., Hou Y., Zhong Y., Meng J., Fan B., Liu L. (2010). Comparative safety evaluation of Chinese pu-erh green tea extract and pu-erh black tea extract in wistar rats. J. Agric. Food Chem.

[19] Yadav S.K., Ahuja P.S. (2007). Towards generating caffeine-free tea by metabolic engineering. Plant Foods Hum. Nutr.

[20] Xu Y., Zhao H., Zhang M., Li C.J., Lin X.Z., Sheng J., Shi W. (2011). Variations of antioxidant properties and NO scavenging abilities during fermentation of tea. Int. J. Mol. Sci.

[21] Wang B.-S., Yu H.M., Chang L.-W., Yen W.-J., Duh P.-D. (2008). Protective effects of pu-erh tea on LDL oxidation and nitric oxide generation in macrophage cells. LWT Food Sci. Technol.

[22] Kumamoto T., Togo S., Ishibe A., Morioka D., Watanabe K., Takahashi T., Shimizu T., Matsuo K., Kubota T., Tanaka K. (2008). Role of nitricoxide synthesized bynitricoxide synthase 2 in liver regeneration. Liver Int.

[23] Mizel S.B., Honko A.N., Moors M.A., Smith P.S., West A.P. (2003). Induction of macrophage nitric oxide production by Gram-negative flagellin involves signaling via heteromeric Toll-like receptor 5/Toll-like receptor 4 complexes. J. Immunol.

[24] Kadowaki S., Chikumi H., Yamamoto H., Yoneda K., Yamasaki A., Sato K., Shimizu E. (2004). Down-regulation of inducible nitric oxide synthase by lysophosphatidic acid in human respiratory epithelial cells. Mol. Cell Biochem.

[25] Davis R.L., Sanchez A.C., Lindley D.J., Williams S.C., Syapin P.J. (2005). Effects of mechanistically distinct NF-κB inhibitors on glial inducible nitric-oxide synthase expression. Nitric Oxide.

[26] Yang Z.Y., Tu Y.Y., Susanne B., Dong F., Xu Y., Naoharu W. (2009). Isolation and identification of compounds from the ethanolic extract of flowers of the tea (*Camellia sinensis*) plant and their contribution to the antioxidant capacity. LWT Food Sci. Technol.

[27] Sava V.M., Yang S.M., Hong M.Y., Yang P.C., Guewha S.H. (2001). Isolation and characterization of melanic pigments derived from tea and tea polyphenols. Food Chem.

[28] Laurentin A., Edwards C.A. (2003). A microtiter modification of the anthrone-sulfuric acid colorimetric assay for glucose-based carbohydrates. Anal. Biochem.

[29] Bradford M.M. (1976). A rapid and sensitive method for the quantitation of microgram quantities of protein utilizing the principle of protein-dye binding. Anal. Biochem.

[30] Livak K.J., Schmittgen T.D. (2001). Analysis of relative gene expression data using real-time quantitative PCR and the 2^−ΔΔCT^ method. Methods.

[31] Schmittgen T.D., Livak K.J. (2008). Analyzing real-time PCR data by the comparative C_T_ method. Nat. Protoc.

